# Analysis Factors That Influence Escalator-Related Injuries in Metro Stations Based on Bayesian Networks: A Case Study in China

**DOI:** 10.3390/ijerph17020481

**Published:** 2020-01-11

**Authors:** Yingying Xing, Shengdi Chen, Shengxue Zhu, Jian Lu

**Affiliations:** 1 College of Transportation Engineering, Tongji University, Key Laboratory of Road and Traffic Engineering of the State Ministry of Education, Shanghai Key Laboratory of Rail Infrastructure Durability and System Safety, Shanghai 201804, China; yingying199004@tongji.edu.cn; 2 School of Transport & Communications, Shanghai Maritime University, 1550 Haigang Street, Shanghai 201306, China; sdchen@shmtu.edu.cn; 3 Jiangsu Key Laboratory of Traffic and Transportation Security, Huaiyin Institute of Technology, Huaian 223003, China; zsx10316@hyit.edu.cn

**Keywords:** escalator-related injury, metro station, risk factors, Bayesian network, probability and severity

## Abstract

Escalator-related injuries have become an important issue in daily metro operation. To reduce the probability and severity of escalator-related injuries, this study conducted a probability and severity analysis of escalator-related injuries by using a Bayesian network to identify the risk factors that affect the escalator safety in metro stations. The Bayesian network structure was constructed based on expert knowledge and Dempster–Shafer evidence theory, and further modified based on conditional-independence test. Then, 950 escalator-related injuries were used to estimate the posterior probabilities of the Bayesian network with expectation–maximization (EM) algorithm. The results of probability analysis indicate that the most influential factor in four passenger behaviors is failing to stand firm (*p* = 0.48), followed by carrying out other tasks (*p* = 0.32), not holding the handrail (*p* = 0.23), and another passenger’s movement (*p* = 0.20). Women (*p* = 0.64) and elderly people (aged 66 years and above, *p* = 0.48) are more likely to be involved in escalator-related injuries. Riding an escalator with company (*p* = 0.63) has a relatively high likelihood of resulting in escalator-related injuries. The results from the severity analysis show that head and neck injuries seem to be more serious and are more likely to require an ambulance for treatment. Passengers who suffer from entrapment injury tend to claim for compensation. Severe injuries, as expected, significantly increase the probability of a claim for compensation. These findings could provide valuable references for metro operation corporations to understand the characteristics of escalator-related injuries and develop effective injury prevention measures.

## 1. Introduction

The urbanization of China and the rapid development of large cities has caused many urban problems such as traffic congestion and air pollution. Metro systems are an important way to improve the efficiency of urban traffic operation, energy conservation, and emission reduction and are the key link to build a high-quality transportation system and implement public transportation priority policy. As of June 2019, a total of 49 cities in China were approved to build urban rail transit, and the metro systems of 39 cities have been put into operation. However, with an increasing number of new lines being brought into service, the safety and reliability of metro operation has become an issue that is of great concern to the public.

Most stations are located underground. In other cases, a station may be elevated above a road; therefore, a large number of escalators, stairs, and elevators must be installed. According to the safety rules for the construction and installation of escalators and moving walks in China, metro stations should use heavy-duty public transportation escalators [1]. Heavy-duty escalators have a longer working life and better safety than ordinary escalators in terms of structure, configuration, and performance [2]. Despite this, the safety of metro escalators cannot be ignored. According to a survey, escalator-related injuries account for 67% of all passenger accidents in the Guangzhou Metro [3]. In other cities, many escalator accidents have also occurred in metro stations, and some of them have resulted in severe injuries. For example, on 5 July 2011, the equipment on the escalator of a metro station on the Beijing Metro Line 4 was faulty; the elevator that was going up suddenly reversed and fell down. The accident caused one death, two serious injuries, and 28 minor injuries [4]. Another case related to metro escalator injuries took place on 2 April 2014 at the Jing’an Temple station in Shanghai Metro, where an escalator reversed during the morning rush hour, resulting in 12 people being slightly injured, and one person was seriously injured.

To reduce the probability of escalator-related injuries, there have been many studies exploring the cause of escalator-related injuries [5,6,7,8,9,10,11,12]. Nicolson (2008) put forward that human error was the most significant contributor to escalator-related injuries [5]. McCann and Zaleski (2013) reviewed the passenger deaths recorded in the escalator survey from 1997 to June 2010, and the analysis results show that more than three-quarters of deaths are caused by falls [7]. Chen and Xian (2016) analyzed 609 escalator injuries of preschool children in Guangdong Province, China, and found that clothes (including slippers, dresses, or backpacks with ropes) are the main cause of escalator-related injuries [8]. More recently, a study conducted by Basir et al. (2018) reported that escalator malfunctions and escalator design are moderately positively associated with the escalator-related injuries (*r* = 0.53, *r* = 0.50, where *r* is Pearson correlation coefficient) [9]. However, most previous studies focused on ordinary escalators, and only a few studies analyzed the characteristics of metro escalator-related injuries [3,13,14,15,16]. For example, Chi et al. (2006) conducted an in-depth study of 194 escalator-related injuries in 2000 at Taipei high-capacity Metro Rapid Transit stations. The results show that most escalator -related injuries are caused by passengers performing other tasks [13]. Li et al. (2016) studied the causes of escalator injuries in a single metro station and found that escalator injuries occur more frequently during intensive periods when the high-speed train arrives, and the location of the escalator injuries is mainly in the lower middle of the upward running escalator at Guangzhou South Railway Station [14]. Xing et al. (2019) analyzed the risk factors of different crowds for metro escalator-related injuries, and the results show that people aged 18–39 are more likely to suffer from escalator injuries when they are not accompanied, while elderly passengers are more likely to be injured because of gradual decline of mental and physical capacities found among the aging [3]. Although useful and revealing, few of these studies have analyzed the severity of the metro escalator-related injuries and analyzed their influencing factors.

The most common methods that are used to analyze escalator-related injuries, such as statistical methods, rely on observed data, covariates, and responses, rather than attempting to simulate the causal process that leads to the adverse outcome [10,13,17,18,19]. The Bayesian network is a good cause–effect analysis tool for representing uncertain knowledge in probabilistic systems and has proven to be effective for capturing and integrating qualitative and quantitative information from various sources [20,21,22]. Moreover, the Bayesian network is able to learn and reason with limited, incomplete, or uncertain information. Berchialla et al. (2016) also found that Bayesian networks both have ease of interpretability and accuracy in predicting probability of body injuries [23].

Therefore, the aim of this study is to understand the characteristics of escalator-related injuries and identify risk factors affecting escalator safety. On the basis of 950 escalator-related injuries that occurred in Guangzhou metro stations in China, a Bayesian network was implemented in order to provide a framework for the probability and severity analysis of escalator-related injuries. The results can be used to develop effective injury prevention measures and document the need for continued improvement of escalator safety in metro stations.

## 2. Materials

**Figure 1:**
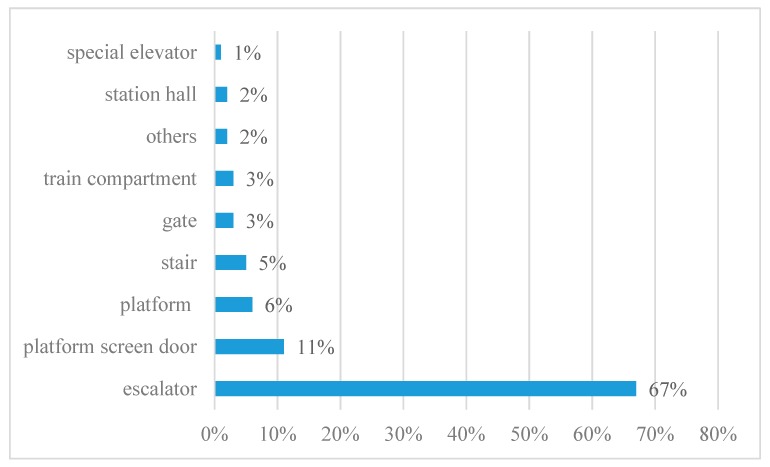
Distribution of passenger injury accidents by the location of the accident.

Metro escalator-related injury data were obtained from the Guangzhou Metro Corporation (GMC). Guangzhou Metro is one of the most representative metro systems in China. It consists of 10 lines and 167 stations. It also ranks third in China by annual ridership after Beijing and Shanghai, with 2.4 billion rides delivered in 2015. However, due to the huge passenger flow, passenger injury accidents occurred frequently in metro stations, such as suicide, being caught in the platform screen door, falls on the escalators, and so on. In terms of the location of accidents, escalator-related injuries account for 67% of all passenger injury accidents, as shown in Figure 1.

Guangzhou Metro Company prepared a case report for each incident and recorded it in the management information system (MIS). Each injury report records the detail of the incident, including the date and the time of the incident, location, the age and gender of the victim, the escalator number, the direction of the escalator, main reason, a detailed description of the incident, and any other factors considered relevant. There were 950 metro escalator-related injuries that occurred on 149 metro stations on 10 lines of the Guangzhou Metro from 2013 to 2015.

## 3. Methodology

### 3.1. Bayesian Network

**Figure 2:**
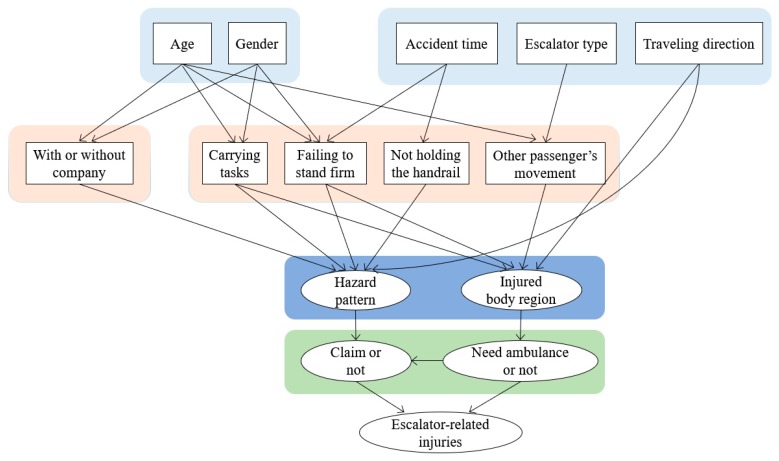
Bayesian network structure based on expert knowledge.

The Bayesian network (BN), also called belief network, was proposed by Pearl in 1985 [24] and combines graph theory and probability theory [25]. In recent years, it has been one of the most effective theoretical models in the field of uncertain knowledge expression and inference. A Bayesian network is actually a directed acyclic graph (DAG) consisting of several nodes representing variables and directed edges reflecting cause–effect relationships of different nodes. In a Bayesian network, if two nodes are connected by a directed edge, it means that one node is the “cause (parent node)”, the other one is the “effect (child node)”. For example, as shown in Figure 2, the node “accident time” points to the node “not holding the handrail”, indicating that not holding the handrail is affected by the accident time. As a result, not holding the handrail is the child node while the accident time is the parent node.

The basic principles of a BN are conditional independence and joint probability distribution. For a BN, the joint probability distribution, P, over the set of variables $V=\left\{V_{1},V_{2},\ldots ,V_{n}\right\}$, can be described as:

(1) $$P\left\{V_{1},V_{2},\ldots ,V_{n}\right\}=\overset{n}{\underset{i=1}{\prod }}P\left(V_{i}|\pi \left(V_{i}\right)\right)$$

where $\pi \left(V_{i}\right)$ represents the parent variables of variable $V_{i}$. The nodes in V are in one-to-one correspondence with the variables $X_{i}$. For a node without any parent nodes, the conditional probability is the same as the prior probability, such as age and gender, as shown in Figure 2.

### 3.2. Dempster–Shafer Evidence Theory

The Dempster–Shafer (DS) theory of evidence was first developed by Dempster [26] and then extended by Shafer [27]. It is a generalization of the Bayesian theory of subjective probability [28]. This theory has proven to be an effective tool applied in expert systems and information fusion. [29,30,31,32].

Let Θ be a finite set including several limited and mutually exclusive elements of a particular proposition, which is called the frame of discernment. The basic functions of Dempster–Shafer evidence theory can be shown as below:

(2) $$m\left(A\right)=\{\begin{array}{c} \frac{1}{1-K}\cdot \underset{A_{1}\cap A_{2}\cap \cdot \cdot \cdot \cap A_{n}}{\sum }m_{1}\left(A_{1}\right)\cdot m_{2}\left(A_{2}\right)\cdot \cdot \cdot m_{N}\left(A_{N}\right),A\neq \emptyset \\ 0,A=\emptyset \end{array}$$

where m(A) is a basic probability assignment (BPA) of Dempster–Shafer evidence theory, which is also the mass function of object A. m(A) is a function of the power set $2^{\Theta }$ to [0, 1], satisfying:

(3) $$\{\begin{array}{c} m\left(\emptyset \right)=0\\ \underset{A\subseteq \emptyset }{\sum }m\left(A\right)=1 \end{array}$$

where ∅ is an empty set, A is any subset of Θ, and $\underset{A\subseteq \emptyset }{\sum }m\left(A\right)=1$ shows that the total value of the reliability of $2^{\Theta }$ needs to be 1. K is the conflict degree of $m_{1},m_{2}\cdot \cdot \cdot m_{N}$, is defined as

(4) $$\begin{array}{cc} K & =\underset{A_{1}\cap A_{2}\cap \cdot \cdot \cdot \cap A_{N}\neq \emptyset }{\sum }m_{1}\left(A_{1}\right)\cdot m_{2}\left(A_{2}\right)\cdot \ldots m_{N}\left(A_{N}\right)\;\\ & =1-\underset{A_{1}\cap A_{2}\cap \cdot \cdot \cdot \cap A_{N}\neq \emptyset }{\sum }m_{1}\left(A_{1}\right)\cdot m_{2}\left(A_{2}\right)\cdot \ldots m_{N}\left(A_{N}\right) \end{array}$$

## 4. Bayesian Network Building

### 4.1. Bayesian Network Nodes

On the basis of extensive analysis of typical escalator-related injuries and further confirmation by expert knowledge, 14 basic nodes with causal relationship were determined. Through this process, the universality of the Bayesian network could be ensured. Table 1 shows the BN nodes of escalator-related injuries and their classifications. The description of each BN node for representing escalator-related injuries is as follows:

#### 4.1.1. Passenger Factor

Human factors are generally considered to be contributing factors on the metro escalator safety. The age and gender of passengers are considered as two nodes in the Bayesian network. According to previous studies, passenger age is divided into five groups, i.e., ≤6, 7–17, 18–40, 41–65, and ≥66 [33,34,35]. Traveling alone or with company describes the state of passengers when taking an escalator and has an effect on the number of injuries. Thus, it should be taken into account by the Bayesian network. There are a variety of passenger behaviors that may contribute to escalator-related injuries, among which four types of high distribution percentage are selected as nodes of the Bayesian network to discuss: Failing to stand firm, carrying out other tasks, not holding the handrail, and another passenger’s movement.

#### 4.1.2. Environmental Factor

Environmental factors include the time when the escalator-related injury occurred and the type and travel directions of escalators. Different occurrence times will cause different numbers of injuries. Based on the working time pattern and people’s lifestyle in Guangzhou, five temporal groups are obtained: Operation opening time–07:29, 07:30–09:29 (morning peak), 09:30–17:29 (working hours), 17:30–19:59 (evening peak), 19:30–operation closing time. It is obvious that escalators have two travel directions, namely upward and downward. A long escalator in Guangzhou Metro is defined as an escalator of more than or equal to 12 m in vertical hoisting height.

#### 4.1.3. Injury Factor

Injury factors involve two nodes: Injured body region and hazard pattern. The injured body regions are grouped into five categories: (1) multiple body regions, (2) head and neck, (3) lower extremity, (4) upper extremity, trunk, and (5) other. Three hazard patterns are considered in this study: Falls, entrapment, and injuries caused by falling objects.

#### 4.1.4. Severity Factor

In the case report provided by the Guangzhou Metro, two factors, whether or not an ambulance was called and whether or not a claim was made, are able to reflect the injury severity of victims to some extent. If an ambulance is needed, it usually means that passengers are seriously injured. In addition, if passengers are badly injured due to the failure of escalators, they may lodge a claim with the metro operation company. According to the records of escalator-related injuries, the claim or lack thereof is classified into five groups: Claim for compensation, have a tendency to claim, reserve the right to claim, no claim, and other unknown situations.

### 4.2. Bayesian Network Structure

With the nodes defined, the causal relationships of all factors contributing to escalator-related injuries were explored by consulting five domain experts. Then, the DS evidence theory was used to synchronize the opinions of the experts to reduce their subjectivity [36]. Nevertheless, there are also cases where experts’ opinions are uncertain, and thus the causal relationship cannot be determined. In this case, the mutual information was applied to determine the relationship between two nodes as it can measure whether the two nodes are dependent and how close their relationship is. For nodes $X_{i}$ and $X_{j}$, the mutual information $I\left(X_{i};X_{j}\right)$ between them is defined as:

(5) $$I\left(X_{i};X_{j}\right)=\underset{X_{i},X_{j}}{\sum }P\left(X_{i},X_{j}\right)\log\frac{P\left(X_{i},X_{j}\right)}{P\left(X_{i}\right)P\left(X_{j}\right)}$$

If $I\left(X_{i};X_{j}\right)$ is greater than a certain threshold ε, there is a causal relationship between $X_{i}$ and $X_{j}$. Given the low probability of escalator-related injuries, the threshold ε=0.015.

**Figure 3:**
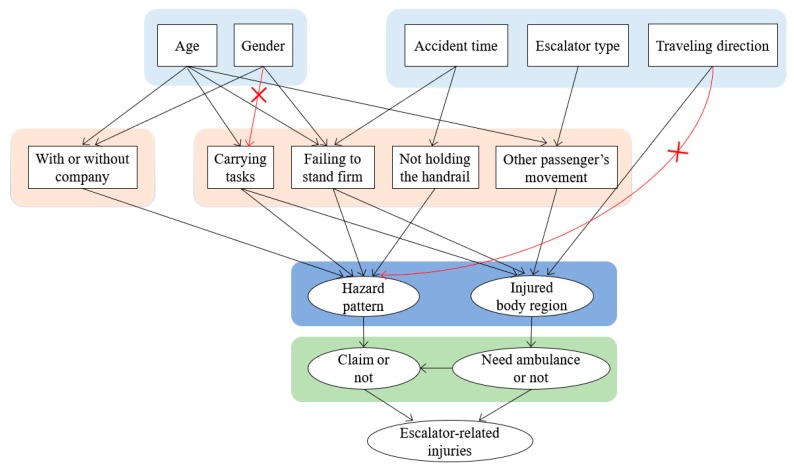
Modification of the Bayesian network structure.

**Figure 4:**
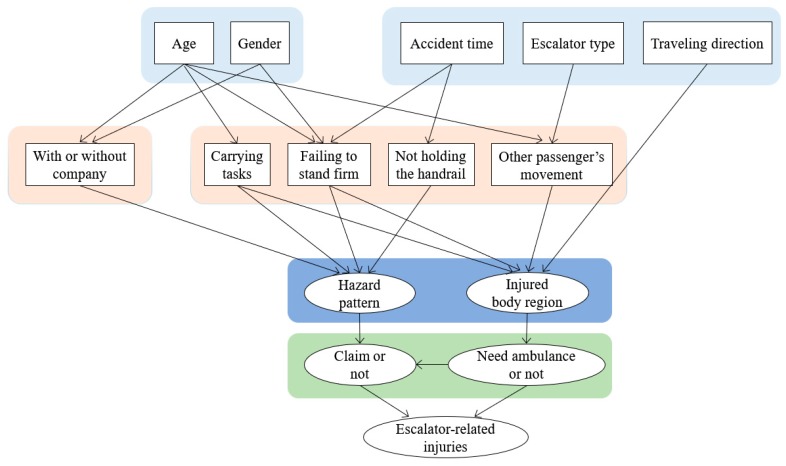
Bayesian network structure based on conditional independence.

Based on the DS theory and the concept of mutual information, the preliminary structure of the Bayesian network is obtained, as shown in Figure 2, but it should be noted that a Bayesian network developed using causal relationships must satisfy the assumption of conditional independence, which may not be considered in this preliminary network structure. To obtain an optimal network structure, a conditional independence test needs to be conducted. In this study, the conditionally mutual information was used to apply conditional independence to a pair of nodes, which is an important and widely used indicator to modify the structure of a Bayesian network [25,37]. The mutual information $I\left(X_{i};X_{j}\right)$ of nodes $X_{i}$ and $X_{j}$ given condition C is defined as:

(6) $$I\left(X_{i};X_{j}|C\right)=\underset{X_{i},X_{j},C}{\sum }P\left(X_{i},X_{j},C\right)\log\frac{P\left(X_{i},X_{j}|C\right)}{P\left(X_{i}|C\right)P\left(X_{j}|C\right)}$$

where C is a set of nodes, and when $I\left(X_{i};X_{j}|C\right)$ is smaller than a certain value, the nodes $X_{i}$ and $X_{j}$ are considered to be independent given C. Given escalator-related injuries with low probability, the threshold value is 0.015. Through the conditional independence test, the arc between factors “gender” and “carrying out other tasks” and “travel direction” and “hazard pattern” were removed permanently, as shown in Figure 3. As a result, the optimal Bayesian network structure for escalator-related injuries was established and is shown in Figure 4.

### 4.3. Learning the Parameters of the Bayesian Network

After the optimal structure of a Bayesian network was established, 950 escalator-related injuries cases were used to estimate the parameters of the BN, i.e., the conditional probabilities or posterior probabilities, which capture the relationships and interplay among the factors and find the influence of the different factors on escalator-related injuries. This process is parameter estimation or “learning” [38,39]. If the training data are complete, it is not difficult to learn BN parameters; however, in the real world, training data may be incomplete for a variety of reasons. For example, when modelling consumer behaviors using a BN, the training data may be incomplete because of privacy issues [40]. In this study, the training data were also incomplete as some useful information that may affect escalator-related injuries was not recorded, such as the speed and gradient of the escalator and the dress and footwear of the victim. For an incomplete dataset, different algorithms have been explored to find maximum-likelihood estimates of parameters (i.e., the posterior probability) for a BN, including expectation–maximization [40,41,42,43,44,45], Markov chain Monte Carlo methods such as Gibbs sampling [46], and gradient descent methods [47]. Among these algorithms, the most popular method is using an EM algorithm [48]. An expectation–maximization (EM) algorithm is an iterative algorithm that converges to a maximum likelihood estimate [45]. It can be divided into two steps: One is E step, which calculates expectation, the other one is M step, which calculates maximum [48]. Previous studies proved that the EM algorithm could be applied in parameter learning of a BN [40,41,42,43,44,45,49]. Therefore, in this study, the EM algorithm was used to estimate posterior probabilities of the BN in the software of Genie 2.0, which is a tool for artificial intelligence modeling and machine learning with Bayesian networks [25]. In this software, the probability of escalator-related injuries was set to 100% because all injuries have already happened. Whether or not an ambulance was called describes the injury severity of escalator-related incidents to a certain extent, as well as whether or not a claim was made. Therefore, these two severity factors were selected to be representatives and set to 100% to explore the impact of various risk factors on the severity of escalator-related injury.

## 5. Results

### 5.1. Probability Analysis of Escalator-Related Injuries

**Figure 5:**
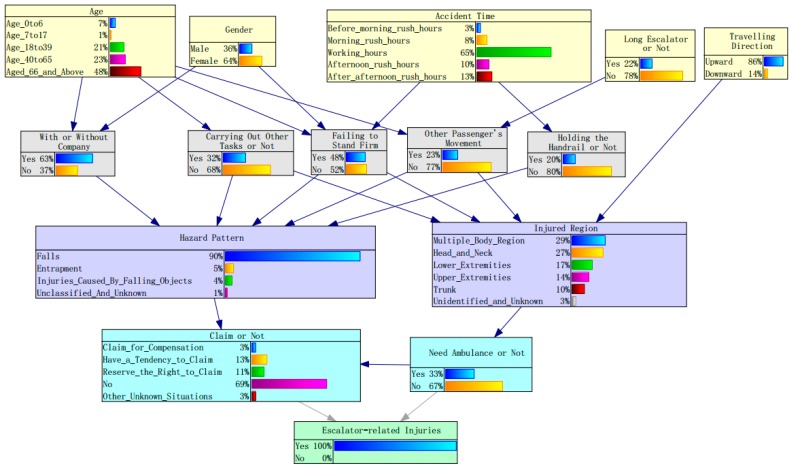
Posterior probabilities for factors contributing to escalator-related injuries.

Figure 5 and Table 2 show the posterior probabilities of the factors that affect escalator-related injuries estimated by Genie 2.0. The posterior probability for female is 0.64, which is significantly higher than male. This finding is in line with the findings in previous studies [3,13,19]. Within the age group, the elderly passengers (aged 65 years and above) have the highest posterior probability (0.48), followed by the middle-aged passengers (0.23). This is probably because the physical and mental states of the elderly gradually decrease with age [50]. Working hours (0.65) are also a significant factor contributing to escalator-related injuries, implying that the large passenger flow during peak hours is not a risk factor for escalator-related injuries.

The posterior probability for the long escalator is 0.22 while the conventional escalator is 0.78, indicating that most escalator-related injuries occur on conventional escalators. However, it should be noted that the long escalators only account for 5.8% of all escalators in metro stations. Compared with this low percentage, the posterior probability of the long escalator being involved injuries is much higher. This suggests that long escalators may be a potential risk factor for escalator-related injuries. Travelling upward exhibits a much higher posterior probability (0.86) than travelling downward. This finding is mainly because the majority of the escalators in Guangzhou Metro are going upward (73.2%).

It is surprising to find that the posterior for riding an escalator with company has a relatively high posterior probability (0.63), which means that passengers are more likely to be involved in escalator riding accidents when they have companions. One possible reason is that elderly passengers (aged 65 years and above) are more prone to be involved in escalator-related injuries and have a greater tendency to travel with company. If an aged passenger fails to stand firm, his/her companion would try to protect them from falling down, which may result in more injuries. Another possible reason may be the distraction and inattention caused by interactions between companions when they take the escalator together. A study on burn injuries in the Netherlands conducted by Hertog et al. (2000) also found that 41% of people said their attention had been distracted for a while in cases where there was somebody around [51]. As a consequence, it is necessary to develop effective countermeasures to stop passengers from becoming absent-minded and make them pay more attention on their escalator riding task.

Of four passenger behaviors in this study, failing to stand firm has the highest posterior (0.48), which is realistic based on the current situation for escalators in metro stations. This is probably caused by the relatively high speed of escalators and carelessness of passengers. For slow-moving elderly people, it may be difficult for them to step on the high-speed escalators. Once they miss their step, it is difficult for them to keep their balance on staggered stairs. Therefore, slowing down the running speed of the escalator during working hours may be an effective way to reduce escalator-related injuries.

Carrying out other tasks, such as carrying luggage and looking after accompanied persons, has the second highest posterior probability (0.32), indicating that carrying out other tasks would distract passengers’ attention and make it difficult for passengers stepping on the escalator fully, especially when bringing large suitcases and baby carriages. Among 214 escalator-related injuries caused by carrying out other tasks, 148 passengers carried luggage, 39 passengers looked after their babies or children, and 12 passengers pushed handcarts or baby carriages. Thus, it can be seen that carrying luggage, especially large luggage, is a main cause of escalator-related injuries. This result is consistent with the previous finding that 13% of 3270 slip and fall injuries happened when the worker was carrying an object [46].

Not holding the handrail and another passenger’s movement have similar posterior probability, namely 0.23 and 0.20, respectively. Despite the records of escalator-related injuries having no detailed information about the reason why passengers don’t hold the handrail, we can infer that one possible reason for this behavior is the use of a cellphone when riding the escalator. After field observation of three stations in Guangzhou metro for a week, we found that nearly 70% of passengers who did not hold the handrail were looking at their smartphones. Thus, the cellphone may be a potential source of risk. Passengers’ movement may have a negative effect on other passengers, especially on long escalators.

Within the hazard pattern group, it is obvious that fall has the highest posterior probability (0.90) while injuries caused by entrapment (0.05) and falling objects (0.04) both have a tiny posterior probability, and the posterior probability for unknown reasons tends to be zero. Therefore, fall is the main hazard pattern of escalator-related injuries in Guangzhou metro, which is consistent with McCann and Zaleski’s (2013) research conclusions [8].

In the category of injured body regions, the multiple body regions have the highest posterior probability (0.29), followed by the head and neck (0.27), indicating that these two groups were the most frequently injured body regions, while trunk has the lowest posterior probability (0.10). The posterior probabilities for lower extremities and upper extremities are 0.14 and 0.10, respectively, implying that extremities also account for a considerable proportion of all escalator-related injuries. The posterior probability for unidentified and unknown regions is 0.03, which means that 3% of escalator-related injuries that injured body regions are still unidentified and unknown.

Regarding injuries treatment, the posterior probability for needing an ambulance is 0.33, illustrating that the escalator-related incidents could cause serious injuries and even death. Although there were no deaths in Guangzhou metro, a teenage boy was killed because of the suddenly changed direction of an escalator in a busy Beijing subway station. Hence, metro operation companies should pay more attention to checking the escalator regularly and providing safe service.

Passengers are not likely to make a claim against a metro operation company as the posterior probability for no claims is 0.69 and for claims is only 0.03. This might be because most escalator-related injuries are caused by individual factors, such as failing to stand firm, carrying out other tasks, and not holding the handrail. In such situations, passengers are less prone to complaint. It is noted that some passengers have a tendency to claim (0.13) and reserve the right to claim (0.12), which also accounts for an appreciable part of all escalator-related injuries.

### 5.2. Severity Analysis of Escalator-Related Injuries

Based on the above Bayesian network, severity analysis of the escalator-related injuries was conducted by using two severity factors, namely “need an ambulance or not” and “claim or not”. If an ambulance is needed, it means that passengers are seriously injured. Therefore, need an ambulance was set as an evidence variable, meaning that the status of “need an ambulance” is considered as 100%. Therefore, the impact of different factors on injury severity could be explored by observing the change of posterior probabilities of other factors when an ambulance is definitely needed. A similar method was also applied to the node “claim or not” to investigate the severity of the escalator-related incidents from the perspective of the metro operation unit. A claim for compensation is generally regarded as a serious incident because it may bring economic loss and have a negative effect on corporate image. Through setting node evidence in Genie 2.0, the posterior probabilities of different factors were estimated by using the EM algorithm.

**Figure 6:**
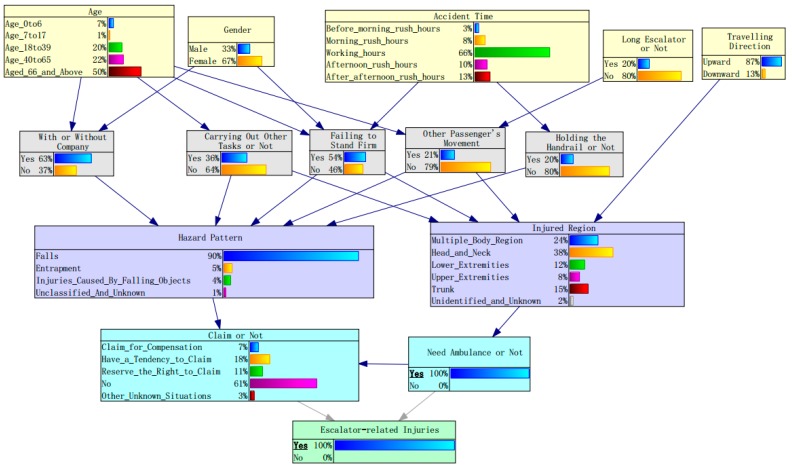
Posterior probabilities for factors contributing to escalator-related injuries on the condition of “need an ambulance”.

The estimated results illustrating the change of posterior probabilities of different contributing factors on the condition of “need an ambulance” are shown in Table 3 and Figure 6. The most significant change of posterior probability happens in injured body regions; the probability for head and neck increases from 0.27 to 0.38, indicating that head and neck injuries are more likely to result in calling an ambulance for treatment. This is probably because the head and neck are the most important and vulnerable parts of the human body. In general, head and neck injuries are more likely to result in very serious consequences. Therefore, head and neck injuries are more likely to urgently need an ambulance than other body injuries. In contrast with head and neck, extremities are less likely to be associated with severe injuries as the decrease of posterior could be observed from both up and down extremities.

Females are more likely to be involved in severe injuries as their posterior probability increases to 0.70. This is probably partially caused by their footwear (in particular for high heels) and since footwear was considered as a risk factor contributing to slipping, tripping, and falling accidents [3,13,52]. Therefore, it is worth collecting passengers’ footwear parameters, such as shoe type, sole, and heel type, to further explore the impact of footwear on escalator-related injuries in future. As expected, the posterior probability of elderly people (aged 66 years and above) rises by 0.03 on the condition of “need an ambulance”, implying that aged passengers are more prone to severe injuries than other age groups.

Within four types of passenger’s behaviors, failing to stand firm has the highest increase of posterior probability (0.06) on the condition of “need an ambulance”, followed by carrying out other tasks (0.04), while another passenger’s movement tends to result in slight injury as its posterior probability change (−0.02) suggests. Therefore, we can conclude that failing to stand firm not only has the most significant effect on injury probability but also tends to result in more serious injuries. After digging deeper into this issue, over 50% of failing to stand firm cases are caused by missing a step or stepping on the junction of two staves when the passengers try to step onto an upward escalator. Therefore, passengers should pay special attention to the yellow line of the moving steps and not step on the yellow line.

Environmental factors, like time of accident and travel direction, seem to have few effects on injury severity. It is surprising to find that long escalators tend to decrease the injury severity as their posterior probability falls by 0.02. One possible explanation is that passengers are more cautious when riding a long escalator.

**Figure 7:**
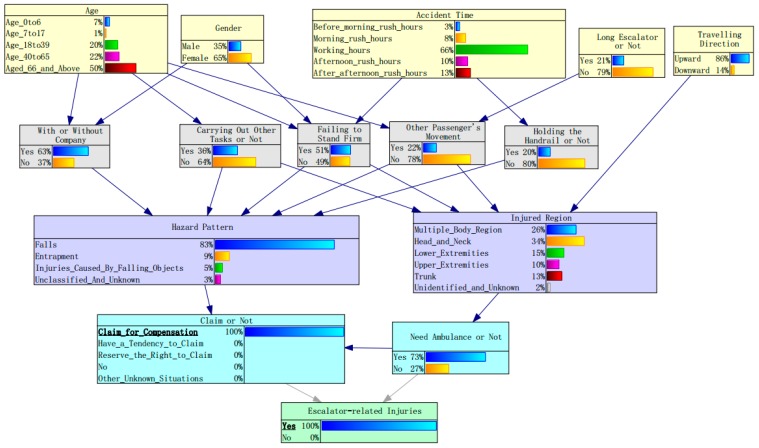
Posterior probabilities for factors contributing to escalator-related injuries on the condition of “claim for compensation”.

Similar results can be observed from the change of posterior probability when “claim for compensation” was set as an evidence variable (see Table 4 and Figure 7). For example, when head or neck are injured, the posterior probability of head and neck increases from 0.27 to 0.34, indicating that the passengers are more likely to claim for compensation in such cases. Compared with men, women have a slightly higher tendency to make a claim to the metro operation corporation. Elderly people (aged 66 years and above) are more likely to claim for compensation than other age groups. Some useful and different conclusions could also be obtained from the estimated posterior probabilities.

Among four types of passenger’s behaviors, carrying out other tasks has the highest increase of posterior probability (0.04) on the condition of “claim for compensation”, followed by failing to stand firm (0.03) and not holding the handrail (0.02), indicating that passengers who are injured due to these behaviors are more likely to lodge a claim. Conversely, another passenger’s movement seems to reduce the probability of a claim because of the decrease of posterior probability (from 0.23 to 0.20). One possible reason is that the injuries caused by another passenger’s movement may not be ascribed to mechanical problems of escalators. Meanwhile, the incidents would generally be negotiated and solved within passengers.

In hazard patterns, passengers who suffer from entrapment injury tend to claim for compensation due to the increase of posterior probability (from 0.05 to 0.09), while the posterior probability of falls is reduced by 0.07. This implies that passengers tend to blame the entrapment injury as it generally results from the direct entrapment of limbs within the gap that exists between the escalator step and the adjacent sidewall, as well as in the escalator comb plate of adjacent staves. In addition, the caught in the escalator (entrapment) accidents generally resulted in more serious injuries than fall [13]. Needing an ambulance has a significant effect on whether or not a claim is made as its posterior probability increases from 0.33 to 0.73, meaning that severe injuries significantly increase the probability of a claim for compensation, which agrees with the expectation.

## 6. Discussion

### 6.1. Strategies for Injury Prevention

This study conducted a probability and severity analysis of escalator-related injuries by using a Bayesian network to identify the risk factors that affect the escalator safety. The results could provide guidance for metro operation corporations to develop effective injury prevention measures. For example, elderly passengers (aged 66 years and above) are involved in a greater proportion of escalator-related injuries and are at higher risk of severe injury. Therefore, metro staff can actively guide elderly passengers to take the elevator instead. However, this preventive approach may not be practical if the elevator provided in the metro station is not easy to access, especially during rush hours.

Failing to stand firm has the highest proportion of escalator-related injuries and tends to result in more serious injuries when compared with other passenger behaviors. This is probably caused by the relatively high speed of escalators and carelessness of passengers. For slow-moving elderly people, it may be difficult for them to step on the high-speed escalators. Once they miss their step, it is difficult to keep the balance on staggered stairs. Since the majority of escalator-related injuries occurred during working hours when the passenger flow pressure is relatively low, slowing down the running speed of the escalator during working hours may be an effective way to reduce escalator-related injuries.

To improve escalator safety, another important approach is to improve the safety awareness of passengers as most escalator-related injuries are mainly caused by individual factors, such as failing to stand firm, carrying out other tasks, and not holding the handrail. The current approach is to remind passengers of the safety guide for an escalator ride with signs and broadcasts, which is not enough. With the popularity of smartphones, new media, such as apps and a WeChat official account, may be a more useful information tool for passengers, especially young and middle-aged people. Moreover, it is easier to accept escalator safety rules by using funny and real pictures instead of boring words. Therefore, escalator safety rules could be presented to children and elderly passengers by using interesting and vivid pictures.

In escalator accidents, head and neck are the most frequently injured body regions and their injuries are more likely to result in very serious consequences. Therefore, the passenger should protect their head to prevent serious injury.

### 6.2. Limitation

This study analyzed the characteristics of escalator-related injuries and identified risk factors affecting escalator safety in metro stations, which is rarely studied but very important to metro operation safety. The useful findings could provide valuable references for metro operation corporations to understand the characteristics of escalator-related injuries and develop effective injury prevention measures. However, there are also some limitations in this study.

Although accident data collected by Guangzhou Metro contain a lot of information, there is still a lack of useful information, such as the speed and gradient of the escalator and the dress and footwear of the victim, which is an intrinsic limitation of this study as these are factors that may have an effect on the probability and severity of escalator injuries. Therefore, the authors suggest that the metro company could record more detailed information for further analysis of escalator-related accidents. In addition, in this study, the severity analysis of the escalator-related injuries was conducted by using two factors, namely “need an ambulance or not” and “claim or not”. Although these two factors could reflect the injury severity of victims to some extent, it needs more quantitative and accurate indicators to measure the severity of escalator-related injuries. Therefore, how to quantify the severity of the escalator-related injuries and analyze their influencing factors deserves further study in future.

## 7. Conclusions

In this study, the Bayesian network was used to identify risk factors that affect escalator-related injuries in metro stations by using 950 escalator-related injuries cases during 2013–2015 in Guangzhou metro. The Bayesian network was developed based on expert knowledge and the DS theory, modified based on the test for conditional independence. The EM algorithm was used to compute the posterior probability of risk factors affecting escalator-related injuries. In addition, by setting “need an ambulance” and “claim for compensation” as an evidence variable, the impact of different factors on the severity of the escalator-related incidents could be obtained.

According to the probability analysis, female passengers (0.64) are more likely to be involved in escalator-related injuries than male passengers, which is consistent with previous studies [3,13]. Elderly passengers (aged 66 years and above, 0.48) are involved in a greater proportion of escalator-related injuries because of gradual decline of mental and physical capacities. Riding an escalator with company (0.63) has a relatively high likelihood to suffer from escalator-related injuries because of the distraction and inattention caused by interactions between companions when they take the escalator together. Of four passenger behaviors, failing to stand firm (0.48) is the most influential factor causing escalator-related injuries, and carrying out other tasks (0.32) has greater influence on escalator-related injuries than not holding the handrail (0.23) or another passenger’s movement (0.20). Falls are the most typical hazard pattern of escalator-related injuries and account for an absolute proportion (0.90). Escalator-related incidents are more likely to result in injuries of multiple body regions, followed by head and neck. About 33% of all the escalator-related injuries require an ambulance to be called, illustrating the serious consequences of escalator-related accidents. Passengers are less likely to make a claim to metro operation corporations as most of escalator-related injuries are caused by individual factors.

The severity analysis shows that head and neck injuries are more likely to require an ambulance to be called for treatment. Female passengers are more likely to be involved in severe injuries, as well as elderly passengers. Failing to stand firm and carrying out other tasks are inclined to result in more serious injuries while another passenger’s movement is less likely to cause severe injuries. Long escalators tend to decrease the injury severity as passengers are more cautions when riding a long escalator. Passengers who suffer from entrapment injury tend to claim for compensation. As expected, severe injuries significantly increase the probability of a claim for compensation.

## Figures and Tables

**Table 1:** Classified states of Bayesian network (BN) nodes.

Bayesian Nodes	States of Nodes
Age	(1) (0–7) (2) (7–17) (3) (17–40) (4) (40–66) (5) ≥66
Gender	(1) Male (2) Female
Accident time	(1) operation opening time–07:29 (2) 07:30–09:29 (3) 09:30–17:29 (4) 17:30–19:59 (5) 19:30–operation closing time
Escalator type	(1) Long escalator (2) Conventional escalator
Traveling direction	(1) Upward (2) Downward
With or without company	(1) With company (2) Without company
Carrying out other tasks or not	(1) Carrying out other tasks (2) Not carrying out other tasks
Failing to stand firm	(1) Failing to stand firm (2) Not failing to stand firm
Holding the handrail or not	(1) Not holding the handrail (2) Holding the handrail
Another passenger’s movement	(1) Yes (2) No
Hazard pattern	(1) Falls (2) Entrapment (3) Injuries caused by falling objects (4) Others including unclassified and unknown
Injured body region	(1) Multiple body region (2) Head and neck (3) Lower extremities (4) Upper extremities (5) Trunk (6) Unidentified and unknown
Claim or not	(1) Claim for compensation (2) Have a tendency to claim (3) Reserve the right to claim (4) No (5) other unknown situations
Need ambulance or not	(1) Yes (2) No

**Table 2:** Posterior probabilities for factors contributing to escalator-related injuries.

Bayesian Nodes		Posterior Probabilities
Age	≤6	0.07
	7–17	0.01
	18–40	0.21
	41–65	0.23
	≥66	0.48
Gender	Male	0.36
	Female	0.64
Accident time	Before 7:30	0.03
	7:30–9:29	0.08
	9:30–17:29	0.65
	17:30–19:29	0.10
	After 19:30	0.13
Escalator type	Long escalator	0.22
	Conventional escalator	0.78
Travel direction	Upward	0.86
	Downward	0.14
With or without company	Yes	0.63
	No	0.37
Carrying out other tasks or not	Yes	0.32
	No	0.68
Failing to stand firm	Yes	0.48
	No	0.52
Another passenger’s movement	Yes	0.23
	No	0.77
Holding the handrail or not	Yes	0.20
	No	0.80
Hazard pattern	Falls	0.90
	Entrapment	0.05
	Injuries caused by falling objects	0.04
	Unclassified and unknown	0.01
Injured body region	Multiple body region	0.29
	Head and neck	0.27
	Lower extremities	0.17
	Upper extremities	0.14
	Trunk	0.10
	Unidentified and unknown	0.03
Claim or not	Claim for Compensation	0.03
	Have a tendency to claim	0.13
	Reserve the right to claim	0.11
	No claim	0.69
	Other unknown situations	0.03
Need an ambulance or not	Yes	0.33
	No	0.67

**Table 3:** Estimated probabilities on the condition of “need an ambulance”.

Bayesian Nodes		Need an Ambulance	
		0.33 ^a^	1 (100%) ^b^
Age	≤6	0.07	0.07
	7–17	0.01	0.01
	18–40	0.21	0.20
	41–65	0.23	0.22
	≥66	0.48	0.50
Gender	Male	0.36	0.33
	Female	0.64	0.67
Escalator type	Long escalator	0.22	0.20
	Conventional escalator	0.78	0.80
Travel direction	Upward	0.86	0.87
	Downward	0.14	0.13
Carrying out other tasks or not	Yes	0.32	0.36
	No	0.68	0.64
Failing to stand firm	Yes	0.48	0.54
	No	0.52	0.46
Another passenger’s movement	Yes	0.23	0.21
	No	0.77	0.79
Injured body region	Multiple body region	0.29	0.24
	Head and neck	0.27	0.38
	Lower extremities	0.17	0.12
	Upper extremities	0.14	0.08
	Trunk	0.10	0.15
	Unidentified and unknown	0.03	0.02

^a^ Posterior probability for needing an ambulance. ^b^ Set evidence = yes.

**Table 4:** Estimated probabilities on the condition of “claim for compensation”.

Bayesian Nodes		Claim for Compensation	
		0.04 ^a^	1 (100%) ^b^
Age	≤6	0.07	0.07
	7–17	0.01	0.01
	18–40	0.21	0.20
	41–65	0.23	0.22
	≥66	0.48	0.50
Gender	Male	0.36	0.35
	Female	0.64	0.65
Escalator type	Long escalator	0.22	0.21
	Conventional escalator	0.78	0.79
Carrying out other tasks or not	Yes	0.32	0.36
	No	0.68	0.64
Failing to stand firm	Yes	0.48	0.51
	No	0.52	0.49
Not holding the handrail	Yes	0.20	0.22
	No	0.80	0.78
Another passenger’s movement	Yes	0.23	0.20
	No	0.77	0.80
Injured body region	Multiple body region	0.29	0.26
	Head and neck	0.27	0.34
	Lower extremities	0.17	0.15
	Upper extremities	0.14	0.10
	Trunk	0.10	0.13
	Unidentified and unknown	0.03	0.02
Hazard pattern	Falls	0.90	0.83
	Entrapment	0.05	0.09
	Injuries caused by falling object	0.04	0.05
	Unclassified and unknown	0.01	0.03
Need an ambulance or not	Yes	0.33	0.73
	No	0.67	0.27

^a^ Posterior probability for claim for compensation. ^b^ Set evidence = yes.
